# Background Noise, Noise Sensitivity, and Attitudes towards Neighbours, and a Subjective Experiment Using a Rubber Ball Impact Sound

**DOI:** 10.3390/ijerph18147569

**Published:** 2021-07-16

**Authors:** Jeongho Jeong

**Affiliations:** Fire Insurers Laboratories of Korea, 1030 Gyeongchungdae-ro, Yeoju-si 12661, Korea; jhjeong92@gmail.com; Tel.: +82-31-887-6737

**Keywords:** rubber ball impact sound, subjective evaluation, background noise, noise sensitivity, attitude toward neighbours

## Abstract

When children run and jump or adults walk indoors, the impact sounds conveyed to neighbouring households have relatively high energy in low-frequency bands. The experience of and response to low-frequency floor impact sounds can differ depending on factors such as the duration of exposure, the listener’s noise sensitivity, and the level of background noise in housing complexes. In order to study responses to actual floor impact sounds, it is necessary to investigate how the response is affected by changes in the background noise and differences in the response when focusing on other tasks. In this study, the author presented subjects with a rubber ball impact sound recorded from different apartment buildings and housings and investigated the subjects’ responses to varying levels of background noise and when they were assigned tasks to change their level of attention on the presented sound. The subjects’ noise sensitivity and response to their neighbours were also compared. The results of the subjective experiment showed differences in the subjective responses depending on the level of background noise, and high intensity rubber ball impact sounds were associated with larger subjective responses. In addition, when subjects were performing a task like browsing the internet, they attended less to the rubber ball impact sound, showing a less sensitive response to the same intensity of impact sound. The responses of the group with high noise sensitivity showed an even steeper response curve with the same change in impact sound intensity. The group with less positive opinions of their neighbours showed larger changes in their subjective response, resulting in the expression of stronger opinions even to the same change in loudness of the impact sound. It was found that subjective responses were different when subjects were performing activities of daily living, such as reading or watching TV in the evening, and when they were focused on floor impact sounds in the middle of the night.

## 1. Introduction

In South Korea, many people go barefoot at home, because they live in apartment buildings with underfloor heating systems. For the rapid construction and supply of apartment buildings, these buildings were built using a load-bearing wall system. Due to the characteristics of the structural system, floor impact sounds and other structure-borne noise is transmitted to neighbouring households via the floors, walls, and other solid structures. In an effort to reduce floor impact sounds, the introduction of floating floor systems became mandatory beginning from 2005, and a prior accreditation system has been enforced to verify the performances of floating floor systems. Nevertheless, differences have been identified between the performances during prior accreditations and the actual performances of floor impact sounds after construction. As such, new strategies are being reviewed to measure and evaluate floor impact sound isolation performances after the construction of apartment buildings. Some researchers have explored the use of heavy/soft impact sources (rubber balls), which have similar properties to the impact sounds actually generated by children running and jumping [1,2,3]. A grading system for rubber ball impact sounds needs to be established in order to use rubber ball impact sounds to evaluate floor impact sound isolation performances in apartment buildings.

When children run and jump or adults walk indoors, the impact sounds conveyed to neighbouring households have relatively high energy in low-frequency bands. The experience of and response to low-frequency floor impact sounds can differ depending on factors such as the duration of exposure, the listener’s noise sensitivity, and the level of background noise in housing complexes. In order to study the responses to actual floor impact sounds, it is necessary to investigate how the response is affected by changes in the background noise and differences in the response when focusing on other tasks. Based on research quantifying the noise sensitivity of individuals to heavyweight floor impact sounds [4], it is necessary to divide subjects into people who are more sensitive or less sensitive to noise and to compare responses between the two groups. Moreover, because floor impact sounds occur in apartment buildings, the responses of residents may differ depending on their relationships with and attitudes towards neighbours. As such, it will be necessary to utilise research on questionnaire-based strategies to define relationships between neighbours [4] in order to study differences in the responses to floor impact sounds.

In this study, the author presented subjects with a rubber ball impact sound recorded from different apartment buildings and housings and investigated the subjects’ responses with varying levels of background noise and when they were assigned tasks to change their level of attention on the presented sound. The subjects’ noise sensitivity and response to their neighbours were also compared. This experiment makes it possible to compare the intensity of rubber ball impact sounds with the intensity of sound reported by the subject in the form of a classification grade. In addition, it is possible to compare differences in the responses depending on the subject’s noise sensitivity and attitudes towards their neighbours.

## 2. Previous Studies

Among the important sources of impact sounds in actual living environments, noise from children running and jumping is known to be the main source [5,6]. Rubber ball impact sounds have been reported to have similar physical and auditory characteristics to the sounds of children running and jumping [7]. Rubber balls have been standardised as heavy/soft impact sources in the international standards [1,2], JIS [8], and KS [9] as a method for measuring floor impact sounds in the laboratory and field conditions. Recently, a simple survey method using rubber ball impact sounds was standardised for on-site quality control [10]. To provide a single number quantity (SNQ) for rubber ball impact sounds, measures such as *L_iA_*_,Fmax_ have been proposed by recording rubber ball impact sounds in apartment buildings made of concrete or wood, measuring subjective responses (e.g., semantic differential), and analysing the correlation between the responses and various single number quantities [11]; these methods were subsequently standardised in ISO 717-2 [3]. A technical specification was standardised in the international standards that defines grades from A to F for air-borne sounds, lightweight impact sounds, and building service equipment-related noise [12]. The ISO TS 19488 standard is based on a study combining actual insulation performances with performance grades in several countries in the European Union (EU COST 9001 [13]) but does not include grades for rubber ball impact sounds.

Grades and methods for evaluating floor impact sounds are being established based on subjective experiments or questionnaire surveys of responses from actual residents in apartment buildings. Jeong [14] investigated the subjective responses of Koreans to lightweight impact sounds and impact sounds from a bang machine and proposed evaluation methods and grades for each source. Kim and Jeon [15] investigated how responses to rubber ball impact sounds were affected by changes in indoor reverberations and reverberation times and by the noise sensitivity of subjects. Ryu et al. [16,17] reproduced heavyweight impact sounds using subwoofers, conducting subjective experiments and measuring the just noticeable difference (JND) in the floor impact sound insulation performance. There have also been subjective experimental studies that have presented floor impact sounds through headphones [18,19] and studies presenting sounds through loudspeakers [19]. The above studies were mostly conducted in a laboratory environment, which differs from the real environments that need to be evaluated. On the other hand, Jeon et al. [20] installed loudspeakers in a laboratory set up similar to real living and working environments and investigated subjective responses to various noises. Jeong [14] investigated the subjective responses in a real housing complex while varying the dropping height of a heavy impact sound source to adjust the sound level.

Since subjective responses to noise vary depending on the individual’s noise sensitivity, noise sensitivity needs to be defined, and evaluation methods need to be established. Weinstein [21] proposed a definition for noise sensitivity and a way of evaluating this using 21 questions. Jeong and Lee [22] administered a questionnaire to assess annoyance and interference with daily life to categorise and compare respondents by their noise sensitivity. Jeong and Lee [23] also administered a questionnaire on annoyance due to the floor impact noise during an 11-year interval; when they analysed and compared the results, they found that the sensitivity to floor impact noise increased with the length of time living in apartment buildings. Park et al. [4] confirmed that noise sensitivity was related to changes in the phycological response to floor impact sounds and traffic noise.

From the research on the relationship between subjective responses of floor impact sounds and psychoacoustic parameters, it was found that the ACF (Auto-Correlation Function) factor, especially for *Φ* (0) and IACC (Inter-Aural Cross-Correlation), had a high correlation. For the lightweight impact sound, *Φ*_1_ correlated well with the subjective responses [24]. Ryu et al. [25] proposed using *Φ*_1_ and τ_1_ for checking the low-frequency tonal signals of air conditioners, ventilation fans, and septic tank pumps. Additionally, they reported that *Φ*_1_ and τ_1_ had a relation with the perception of low-frequency noise. 

## 3. Subjective Experiment Planning

### 3.1. Scope

In this study, the author used a subwoofer and 4 loudspeakers to reproduce rubber ball impact sounds in a subjective experiment laboratory set up to similar to an actual household in a typical apartment building in South Korea. After listening to each sound, subjects were asked to select the grade they thought the sound corresponded to among the grades A–F in ISO/TS 19488 (see Table 1).

The three experiments were performed under the following conditions: an environment with very low background noise, an environment with background noise, and an environment with background noise where the subject was performing the task of browsing internet articles. These three conditions were compared in Table 2. In addition to the subjective experiment, the subjects’ noise sensitivity and attitudes towards neighbours were surveyed using questionnaires.

### 3.2. Participants

The subjects in the subjective experiment consisted of 21 male or female individuals aged 30–59 years living in Seoul or Gyeonggi-do and either currently living in or with experience of living in an apartment building. The subjective experiment was performed 3 times, as shown in Table 2, varying the presence or absence of background noise and having the subject browse the internet to affect their level of attention on the rubber ball impact sounds. To minimise the uncertainty from the number of subjects, the author conducted the experiments on the same subject group.

In order to obtain the subjects’ responses to the reproduced rubber ball impact sounds, the explanation of the classification grades in ISO TS 19488 Annex B was translated into Korean, colour information was added to aid understanding, and the explanation was presented to the subjects (see Table 1). Before the main experiment, a pilot experiment was performed so that the subjective experiment procedure and the volume of the impact sounds could be experienced. The presentation order of the sound stimuli was randomised for each participant and experiment. The experiment was conducted on one subject at a time and lasted a total of 25 min.

In Experiment 3, the subjects were asked to browse the internet or search for and read articles while the background noise was played. Since sounds that occurred while browsing the internet could affect the results of the subjective experiment, only text and graphic information was provided. Each subject’s responses to each sound stimuli were quantified. The averaged subjective responses of each impact sound stimuli were plotted with the impact sound pressure level.

The experiments with different background sound conditions with or without a task were performed at intervals of 2 to 3 weeks. For investigating the noise sensitivity and attitude on the neighbours of each subject, the questionnaire was composed of 21 questions on noise sensitivity and 6 questions on attitude toward the neighbours [21,22,26]. Questions on noise sensitivity and attitude toward the neighbours are in Appendix A.

**Figure 1:**
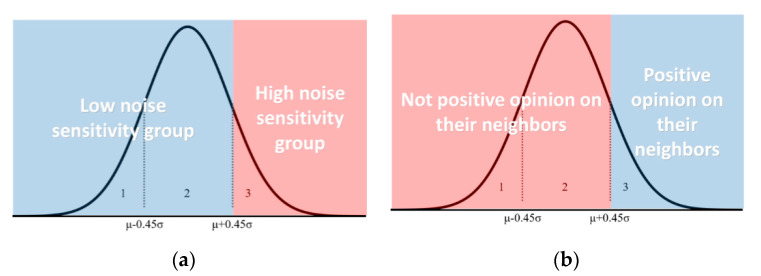
Two subject groups divided based on the questionnaire survey results on noise sensitivity and attitude toward the neighbours. (**a**) Noise sensitivity. (**b**) Attitude toward their neighbours.

For the noise sensitivity, a 6-point scale was used, and a 5-point scale was used for investigating the attitude toward their neighbours. Jeong and Lee [22] surveyed the noise sensitivity and attitude toward their neighbours of 223 Korean participants. As shown in Figure 1, the subjects were divided into two groups based on the population of the previous questionnaire survey results on noise sensitivity and attitude toward their neighbours [22]. Subjective responses of the two groups for the low-frequency rubber ball impact sound were analysed and compared.

### 3.3. Impact Sounds

For the subjective experiment using rubber ball impact sounds, the sounds were recorded from 9 apartment building complexes built from load-bearing wall-type reinforced concrete (RC) structures using a resilient material for the isolation of floor impact sounds, which is the most common construction method in South Korea. In addition, in order to include rubber ball impact sounds in wooden buildings, 6 types of rubber ball impact sounds recorded from wooden housings in Japan [16] were used. Using a sound editing program (Adobe Audition 3.0, Adobe, San Jose, CA, USA), the sound pressure of the 15 types of rubber ball impact sounds was adjusted to create sounds at 5-dB intervals. Each recording was edited with 6 or 7 steps separately. The sound pressures presented to subjects were in the range of 20–67 dB of A-weighted maximum impact sound pressure level *L*_i,A,Fmax_. The A-weighted maximum impact sound pressure level was recently standardised as the single number quantity of a rubber ball impact sound in ISO 717-2:2020 [3]. In total, 104 impact sound stimuli were presented to the subjects.

**Figure 2:**
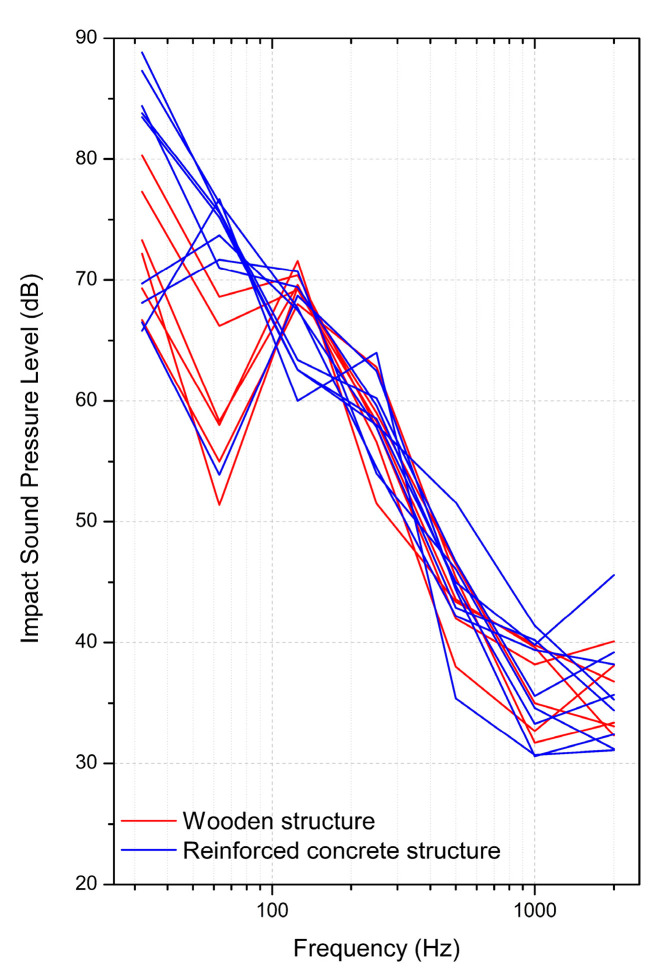
Frequency characteristics of the 15 rubber ball impact sounds used in the subjective experiment.

Figure 2 shows the frequency characteristics of the 15 rubber ball impact sounds in the octave bands. As demonstrated by Figure 2, the impact sounds presented to the subjects showed the highest sound pressure in the range below the 125-Hz band and showed very low sound pressure above 1000 Hz. The 15 rubber ball impact sounds can be divided into those that show continuously increasing sound pressures at lower frequencies and those that show a sound pressure peak at the 125-Hz band. Among the 15 rubber ball impact sounds, 6 were recorded from wooden housing complexes, and 9 were recorded from RC housing complexes. Figure 2 shows the characteristics of the sounds recorded from wooden structures and those recorded from RC structures separately with different colours. As can be seen in the blues lines in Figure 2, the rubber ball impact sounds recorded from RC structure apartment buildings usually show the characteristics of increasing sound pressures and lower frequencies. One rubber ball impact sound from the RC structure had different frequency characteristics, which had the highest level in the 125-Hz band. The apartment unit where the rubber ball impact sound was recorded had a relatively small indoor space and 60-mm-thick resilient materials.

On the other hand, the rubber ball impact sounds recorded from wooden housing usually showed lower sound pressure at the 63-Hz band than at the 125-Hz or 32-Hz bands (see the red lines in Figure 2). The author also analysed and compared the differences in the subjects’ subjective responses due to the discrepancy in the sound pressure at the 63-Hz band between the rubber ball impact sounds recorded from each type of housing.

### 3.4. Equipments and Environment

In order for the subjects to experience the 15 rubber ball impact sounds as if they were in a real apartment building, a subwoofer (GENELEC 7060B Active subwoofer, GENELEC, Iisalmi, Finland) was used to reproduce the impact sounds in the low-frequency range. In addition, in order to reproduce the feeling of real impact sounds being produced from the ceiling, 4 loudspeakers (GENELEC 8030A Amplified monitor speaker, GENELEC, Iisalmi, Finland) were placed in the upper part of the laboratory and operated alongside the subwoofer. The speaker system was connected to a PC using an AD converter (MOTU 896, MOTU, Cambridge, MA, USA).

**Figure 3:**
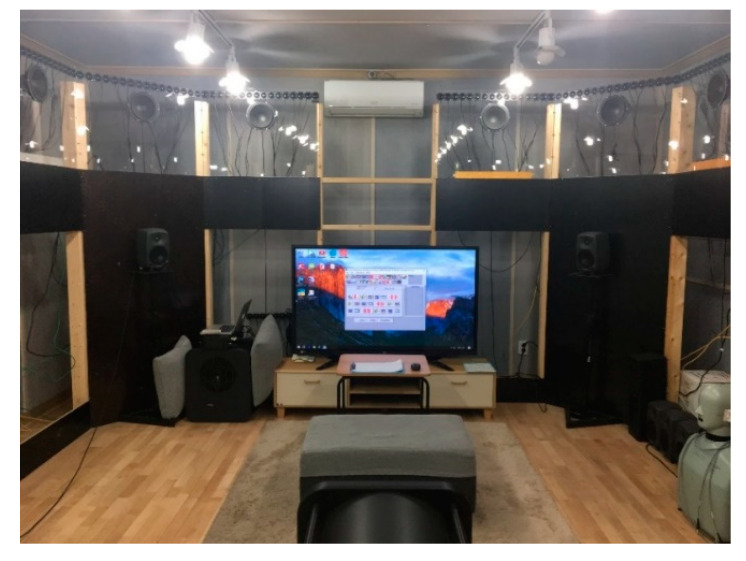
Listening chamber where the subjective experiment was conducted.

For subjects to input their responses, in order to recreate the feeling of watching TV in the living room of an actual housing complex, a large TV was placed in the subjective experiment booth and connected to a PC, so that subjects could input their responses while watching the TV screen. The subjective experiment was conducted in a booth (7.5 m × 3.5 m × 2.4 m; see Figure 3) with around 20 dBA of background noise. The booth was furnished with a sofa, rug, and TV similar to the living room in a typical household in South Korea (see Figure 3). The average reverberation time from the 500-Hz band to the 2000-Hz band of the experimental environment was 0.28 s, which was similar to or a little shorter than the reverberation time in typical housing [27].

In Experiments 2 and 3, which were conducted in the presence of background noise, pink noise was played through a half omnidirectional loudspeaker. The loudspeaker was placed on the floor of the booth so that the subjects were unaware that a speaker was being used to produce background noise. In the background noise experiments, the pink noise was started before the subject entered the subjective experiment booth, so that the subject could not differentiate when the background noise was being played.

## 4. Results of the Subjective Experiment

**Figure 4:**
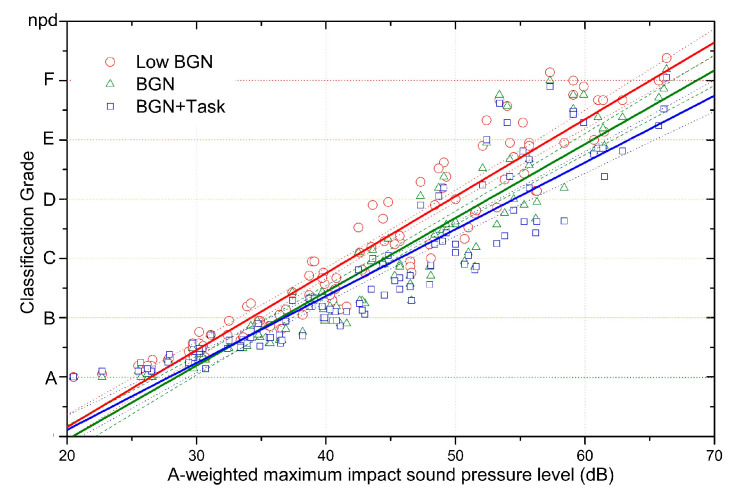
Changes in the subjective responses with varying impact sound pressure levels in the presence or absence of background noise and with the addition of an internet browsing task. Dashed and dotted lines show the 95% confidence levels of the regression line.

**Figure A1:**
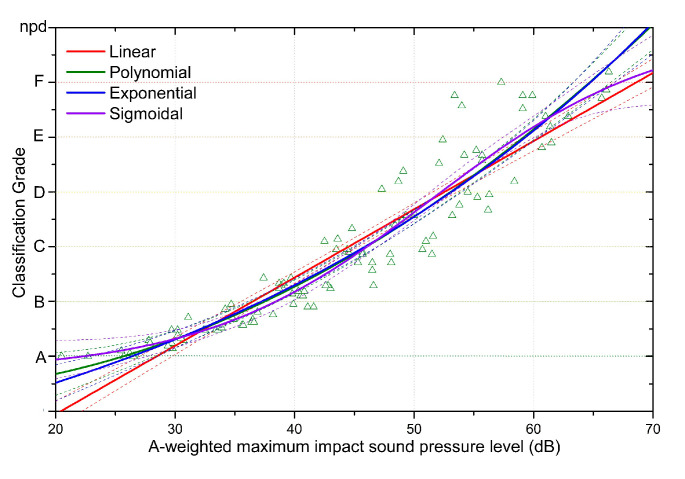
Regression lines and the 95% confidence levels of each regression lines on the responses of the 2nd experiment.

The average values of the participants’ responses were plotted with *L*_i,A,Fmax_ in Figure 4. The responses were not perfectly linear. To compare the responses of participants from each experiment, regression methods were adopted and compared (see Appendix B, Figure A1 and Table A1). The polynomial and exponential regression methods showed higher R-square values than the linear regression method. However, previous studies [16,28,29,30,31,32,33] on the subjective evaluation of floor impact sounds adopted the linear regression analysis. In this study, the subjective experiment results were analysed using a linear regress analysis to maintain consistency with previous studies. In addition, the 95% confidence level of the linear regression lines of each regression line were plotted with dashes with the same colour as the regression line in each figure.

### 4.1. Effects of Background Noise and Task Performance on Responses to Rubber Ball Impact Sounds

Figure 4 shows the subjective responses to the same rubber ball impact sounds for each of the background noise and task conditions. *L*_i,A,Fmax_, which has been standardised in ISO 717-2:2020 Annex D, was used for the presented sounds. A linear regression analysis was performed on the subjective responses for each condition, and the regression lines were added to Figure 4. The results of the linear regression analysis for each condition are summarised in Table 3.

In the condition with very little background noise and the condition with the background noise controlled at around 37 dB (A), the subjective responses to the rubber ball impact sounds in each of the two environments grew further apart as the presented sound pressure level increased. Comparing the slopes from the linear regression equations in Table 3, the slope was 0.12974 in the low background noise condition and 0.1245 in the condition with background noise, meaning that the subjective responses changed more in the condition with low background noise. The slope was 0.1127 when the subjects also browsed the internet in the presence of background noise, representing the smallest change in subjective response for the same differences in sound pressure levels. The R-square value of the subjective responses was highest in the condition with low background noise and decreased sequentially with the addition of background noise and internet browsing. These findings indicate that background noise and performing a task such as internet browsing interfered with the subjects’ attention and consistent response to rubber ball impact sounds.

Based on the subjective responses and the results of the linear regression analysis in each of the 3 experimental conditions, the *L*_iA,Fmax_ values corresponding to the ISO/TS 19488 classification grades were calculated; these are summarised in Table 4. When comparisons were made based on Class D, which is indicated as the requirement for new buildings in ISO/TS 19488, from the linear regression analysis results, in the low background noise condition, Class D was estimated to be 49.6 dB, in the condition with background noise added, Class D was estimated to be 52.6 dB, and in the condition with background noise and internet browsing, Class D was estimated to be 54.5 dB. Thus, the change in the subjective responses due to background noise was around 3 dB for Class D-level noises, and the change when performing a task like browsing the internet was around 1.9 dB.

In the 3 experimental conditions, the rubber ball impact sound levels corresponding to Class B were 34.2 dB, 36.5 dB, and 36.8 dB, meaning that the change in the subjective responses due to background noise was 2.3 dB, and the change when browsing the internet was 0.3 dB. In other words, when a quieter rubber ball impact sound is presented, background noise and task performance have smaller effects on the subjective response.

### 4.2. Effects of Noise Sensitivity on Rubber Ball Impact Sound Responses

**Figure 5:**
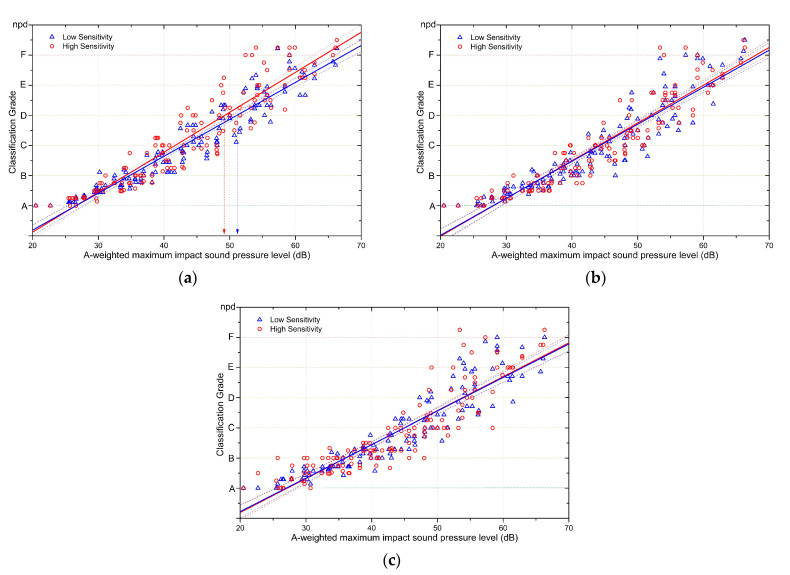
Comparison of the responses to rubber ball impact sounds between the sensitivity groups. Dashed lines show the 95% confidence levels of the regression line. (**a**) Impact sound responses of the two sensitivity groups in the low background noise condition. (**b**) Impact sound responses of the two sensitivity groups in the presence of background noise. (**c**) Impact sound responses of the two sensitivity groups when performing an internet browsing task in the presence of background noise.

Based on the results of the noise sensitivity questionnaire that was administered alongside the subjective experiment, the subjects who participated in the experiment were divided into a high noise sensitivity group and a low noise sensitivity group, as shown in Figure 1a. Figure 5 shows the comparison of the subjective responses to rubber ball impact sounds between the two noise sensitivity groups. Based on these responses, a linear regression analysis was performed in each group, and the regression lines were added to Figure 5. The linear regression equations for each group and condition are summarised in Table 5.

Figure 5a and Table 5 show the responses of each group to rubber ball impact sounds in the low background noise condition. The slope of the subjective response relative to the rubber ball impact sound level was 0.13267 in the high sensitivity group and 0.122264 in the low sensitivity group, meaning that the responses in the high sensitivity group changed more steeply even for the same change in the rubber ball impact sound level. When background noise was added or the task of browsing the internet was added, there was a narrowing of the difference in subjective responses between the sensitivity groups (see Figure 5b,c).

Table 6 shows the estimated classification grades for rubber ball impact sounds based on the linear regression analysis of the results for each of the sensitivity groups. In the low background noise condition, the sound pressure level corresponding to Class D was 51.2 dB in the low sensitivity group and 49.2 dB in the high sensitivity group, meaning that there was a difference of 2 dB due to noise sensitivity. Table 7 summarises the differences between the sensitivity groups in the classification grades for rubber ball impact sounds based on the regression equations for subjective responses in each group. As shown in Table 7, the difference in the classification grade estimates between the two sensitivity groups narrowed as the rubber ball impact sound level decreased. In addition, the difference in subjective responses between the sensitivity groups narrowed when the level of background noise increased and when the subjects had to attend to another task, such as browsing the internet.

In Table 8, the effects of background noise and the internet browsing task were calculated based on the results for the three experimental conditions. The effects of background noise and the task were greater in the group with higher noise sensitivity, and these effects changed more rapidly with varying impact sound levels in the group with lower noise sensitivity.

### 4.3. Effects of Attitudes towards Neighbours on Rubber Ball Impact Sound Responses

**Figure 6:**
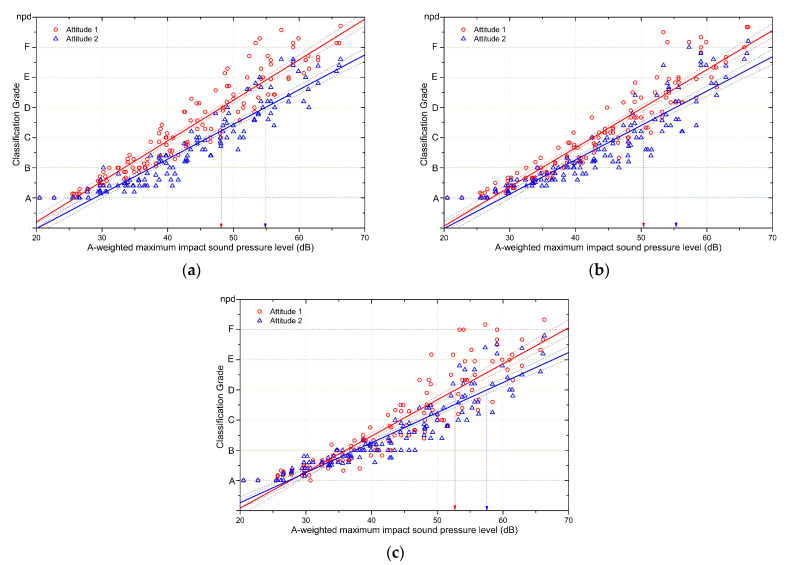
Comparison of the responses to rubber ball impact sounds between the attitude groups. Dashed lines show the 95% confidence level of the regression line. (**a**) Impact sound responses of the two attitude groups in the low background noise condition. (**b**) Impact sound responses of the two attitude groups in the presence of background noise. (**c**) Impact sound responses of the two attitude groups when performing an internet browsing task in the presence of background noise.

Alongside the subjective experiment on rubber ball impact sounds, the subjects’ attitudes towards their neighbours were also investigated. Based on the results, as shown in Figure 1b, the 21 subjects were divided into a group with positive attitudes towards their neighbours and a group without positive attitudes towards their neighbours. A linear regression analysis was performed on the subjective responses for each of the attitude groups. Figure 6 shows the comparison between the two groups under the different experimental conditions with varying levels of background noise and the performance of an internet browsing task. Table 9 shows the linear regression equations for subjective responses in each of the attitude groups.

When the subjective responses to rubber ball impact sounds were compared between the attitude groups in the condition with low background noise, as shown in Figure 6a, there were large differences between the groups. The difference between the groups was larger for louder rubber ball impact sounds, and the difference narrowed as the sound pressure level decreased. The slope for the change in subjective responses relative to the rubber ball impact sound level was 0.1153 in the group with positive attitudes towards their neighbours and was 0.13451 in the group without positive attitudes towards their neighbours. This means that the subjects with positive attitudes towards their neighbours showed less change in their responses to noise. The noise level corresponding to Class D, which was estimated based on the subjective responses, was 48.2 dB for the group without positive attitudes towards their neighbours and was 54.9 dB in the group with positive attitudes, meaning that positive attitudes towards neighbours were associated with a 6.7-dB difference (see Table 10). The difference between the two groups in the sound level corresponding to Class B was 4.2 dB, showing that attitudes towards neighbours had a lesser effect on the responses at lower impact sound levels.

When the two groups were compared in the experimental condition with added background noise, a similar pattern was observed to the condition without background noise. However, although the slopes for the subjective responses became flatter in the presence of background noise, there were some differences in the slopes of each group at 0.114 (positive attitude) and 0.12979 (not positive attitude). The sound pressure level corresponding to Class D in the background noise condition was 50.3 dB in the group without positive attitudes towards their neighbours and 55.2 dB in the group with positive attitudes towards their neighbours, meaning that the difference between the groups was 4.9 dB (See Table 11). When the effects of background noise on the subjective responses were compared between the groups, the group with positive attitudes towards their neighbours showed a difference of 0.3 dB due to background noise, whereas the group without positive attitudes towards their neighbours showed a difference of 2.1 dB due to background noise (see Table 12).

Figure 6c shows the subjective responses in the condition with background noise and internet browsing. The difference in the subjective responses between the two attitude groups decreased further with the addition of the internet browsing task. As shown in Table 9, the slopes for the subjective responses became flatter in both groups when they were browsing the internet. The sound pressure level corresponding to Class D was 57.5 dB in the group with positive attitudes towards their neighbours and 52.8 dB in the group without positive attitudes towards their neighbours, meaning that the difference between the groups was 4.7 dB (see Table 10 and Table 11). As shown in Table 12, the task effect when performing a task like internet browsing was not different between the two groups at 2.3 dB (positive attitude) and 2.5 dB (not positive attitude). This was smaller than the difference between the two groups in the effects of background noise on subjective responses to rubber ball impact sounds.

### 4.4. Effects of Construction Materials Structural Type on Rubber Ball Impact Sound Responses

In the subjective experiment, the sounds presented to subjects consisted of nine types of rubber ball impact sounds recorded from RC structures in South Korea and six types of rubber ball impact sounds recorded from wooden housings in Japan, which had their sound pressure levels adjusted. As shown in the frequency characteristics in Figure 2, the rubber ball impact sounds recorded from the two different structure types showed different characteristics in the below-100-Hz frequency band. Most of the impact sounds recorded from RC apartment buildings showed a linear decrease in sound pressure with increasing frequency, whereas the impact sounds recorded from wooden housings were characterised by lower sound pressure at 63 Hz than at 32 Hz or 125 Hz. Subjective responses to rubber ball impact sounds from each of these structure types were compared with their different acoustic properties.

**Figure 7:**
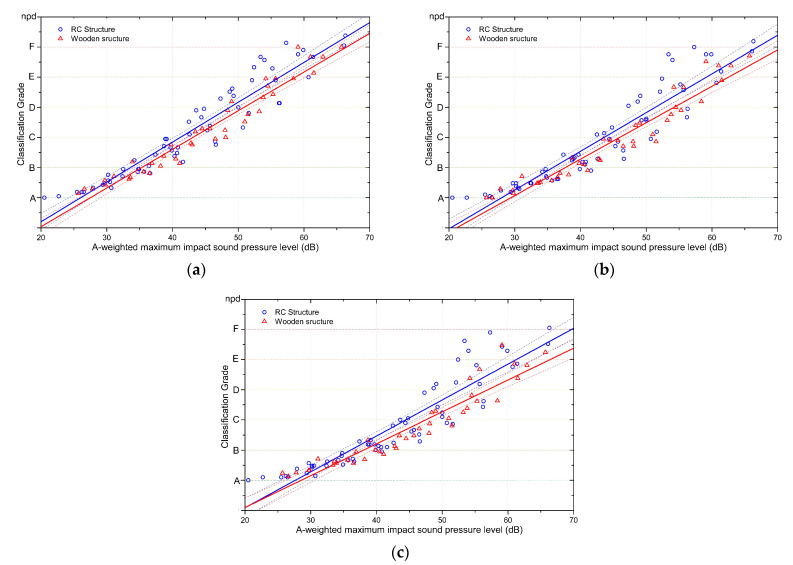
Comparison of responses to rubber ball impact sounds between the structure types. Dashed lines show the 95% confidence levels of the regression line. (**a**) Impact sound responses for the two structure types in the low background noise condition. (**b**) Impact sound responses for the two structure types in the presence of background noise. (**c**) Impact sound responses for the two structure types when performing an internet browsing task in the presence of background noise.

Figure 7 shows the results of the linear regression analysis on the subjective reactions of subjects to impact sounds in each of the structure types under the three different experimental conditions. When background noise and an internet browsing task were added sequentially to the experiment, the graphs of the subjects’ responses relative to the rubber ball impact sound level showed different slopes in the two different structure types.

Moreover, the difference in the responses between these two groups gradually widened with the addition of the background noise and internet browsing conditions. Table 13 shows the regression equations for the subjective responses against the rubber ball impact sound levels in the two structure types. The slope, showing the change in the subjective responses with varying impact sound levels, was steeper in the RC structure group than in the wooden structure group.

Based on the above regression equations, the sound pressures corresponding to the rubber ball impact sound classification grades were calculated and are displayed in Table 14. The sound level corresponding to Class D, which is suggested as the standard for newly built housing complexes, was 54.2 dB in wooden structures in the presence of background noise and 51.4 dB for RC structures. This means that the difference between the two structure types in the subjective responses corresponding to Class D was around 2.8 dB (see Table 15). In the condition with low background noise, this difference (for the Class D level) decreased to 2.2 dB, but in the condition with background noise and an internet browsing task, the difference in the subjective responses between the two structure types increased to 4.1 dB.

Using the classification grades estimated from the linear regression equations of the subjective responses to rubber ball impact sounds, the effects of background noise and the internet browsing task were calculated and are shown in Table 16. The effects of background noise on the subjective responses were greater in wooden housings, and the effects of the internet browsing task were also greater in wooden housing complexes. The reason for the weaker background noise and task effects in RC apartment buildings is thought to be because the rubber ball impact sounds in these structures showed higher sound pressure levels at 63 Hz compared to the impact sounds from wooden structures.

## 5. Conclusions

### 5.1. Results

In this study, the author presented subjects with a rubber ball impact sound recorded from different apartment buildings and housings and investigated the subjects’ responses with varying levels of background noise and when they were assigned tasks to change their level of attention on the presented sound. The subjects’ noise sensitivity and attitudes towards their neighbours were also compared. This experiment makes it possible to compare and validate the evaluation methods for rubber ball impact sounds that show a strong correlation between the actual impact sound level and subjects’ responses in the form of classification grades. It is also possible to compare the impact sound levels that correspond to each grade. Moreover, the effects of the subjects’ noise sensitivity and attitudes towards neighbours on their responses can be investigated.

A subjective experiment using rubber ball impact sounds was repeated three times in a laboratory set up to resemble real housing complexes in South Korea. The participants in the experiment consisted of 21 persons aged 30–59 years who lived in housing complexes. The experiment was performed under three different conditions: low background noise, background noise, and having the subjects perform an internet browsing task in the presence of background noise. The results were compared between the three conditions. In addition, the subjects’ noise sensitivity and attitudes towards neighbours were investigated through questionnaires.

In the results of the subjective experiment, the subjective responses differed depending on the level of the background noise, and this difference in the subjective responses increased with louder rubber ball impact sounds. In addition, performing a task like internet browsing reduced the attention to the rubber ball impact sound, which resulted in a less sensitive reaction to the same level of impact sound.

The subjects were divided into two groups based on noise sensitivity, and the groups were compared. For the same change in the rubber ball impact sound level, the high noise sensitivity group showed a steeper change in their subjective responses. However, the difference in subjective responses between the two sensitivity groups was found to decrease with the addition of background noise and the task that is distracting their concentration on the floor impact sound, internet browsing. Based on the questionnaire about attitudes towards one’s neighbours, the author divided the subjects into those with positive attitudes and those with nonpositive attitudes and compared the subjective responses in the two groups. The change in the subjective responses was greater in the group with nonpositive attitudes towards their neighbours, indicating that these subjects displayed stronger opinions even for the same change in the rubber ball impact sound level. In the condition with very low background noise, the rubber ball impact sound level corresponding to Class D, in terms of the *L*_iA,Fmax_, was about 55 dB in the group with positive attitudes towards their neighbours and was about 48 dB in the group with nonpositive attitudes towards their neighbours, which was a difference of 7 dB. The difference in subjective responses between the two attitude groups decreased to around 5 dB with the introduction of background noise and additional operational demands.

In an analysis of the frequency characteristics of the rubber ball impact sounds presented to the subjects, the sounds from RC housing complexes and the sounds from wooden housing complexes showed different characteristics in the low frequency range. When differences in the subjective responses for each structure type were compared, the subjects showed a relatively weaker response to the rubber ball impact sounds from wooden housings.

### 5.2. Discussions and Future Work

Based on the results of the subjective experiment using rubber ball impact sounds, different goals for impact sound reduction will need to be established depending on the level of background noise in housings. Different subjective responses were observed depending on whether the subject was performing activities of daily living, such as reading or watching TV in the evening, or was focusing on the floor impact sounds in the middle of the night. In addition, differences in noise sensitivity and attitudes towards their neighbours were also associated with different subjective responses. Maintaining cordial relations with one’s neighbours might even reduce dissatisfaction or complaints about floor impact noises.

The results of this paper can be used as reference materials for a reasonable and amicable resolution in the case of a dispute or disputes between neighbours over floor impact sounds. It can be used for the establishment and standardisation of performance standards for rubber ball impact sounds in the future. In addition, to increase the subjective satisfaction concerning floor impact sounds, research on the psychoacoustic parameters and ACF factors of low-frequency impact sounds should be conducted in the future.

## Figures and Tables

**Table 1:** Description terms of the quality of the different classes in ISO/TS 19488.

Class	General
A	A quiet atmosphere with a high level of protection against sound. This class may be applied where a considerably better climate is asked for.
B	Under normal circumstances a good protection against sound without too much restriction to the behaviour of the occupants. This class may be applied where a better acoustic climate is asked for.
C	Protection against considerable distribution under normal behaviour of the occupants, bearing in mind their neighbours. Newer building construction in many countries are likely to fulfil or exceed this class.
D	Disturbance by noise may be expected more than occasionally, even in case of comparable behaviour of occupants, adjusted to neighbours. Newer building construction in most countries are likely to fulfil or exceed this class.
E	A low protection is offered against intruding sounds. To be applied mainly for classification of existing housing (before renovation).
F	A very low protection is offered against intruding sounds. To be applied only for classification of older, existing housing (before renovation)
npd	No performance determined

**Table 2:** The experimental conditions.

Experiment	Background Noise	Internet Surfing
1st	20 dB(A)	-
2nd	Pink noise 37 dB(A)	-
3rd	Pink noise 37 dB(A)	Text based contents

**Table 3:** Linear regression analysis results for the subjective responses during the 3 experimental conditions.

Experiment	Intercept	Slope	R-Square
1st Low-BGN	−2.43833	0.12974	0.90992
2nd With BGN	−2.54295	0.1245	0.87815
3rd With BGN + Task	−2.14434	0.1127	0.84868

**Table 4:** Classification grades calculated based on the subjective experiment results (*L*_i,AFmax_, (dB)).

Experiment.	A	B	C	D	E	F
1st Low BGN	26.5	34.2	41.9	49.6	57.3	65.0
2nd With BGN	28.5	36.5	44.5	52.6	60.6	68.6
3rd With BGN + Task	27.9	36.8	45.6	54.5	63.4	72.3

**Table 5:** Results of the linear regression analysis for subjective responses in each of the 3 experimental conditions for the groups divided by noise sensitivity.

Experiment	Noise Sensitivity	Intercept	Slope	R-Square
1st Low-BGN	High	−2.53086	0.13267	0.86544
	Low	−2.26499	0.122264	0.90348
2nd With BGN	High	−2.51513	0.12525	0.86365
	Low	−2.43993	0.12288	0.85882
3rd With BGN + Task	High	−2.05441	0.11247	0.80495
	Low	−2.00145	0.11117	0.84869

**Table 6:** Rubber ball impact sound classification grades calculated based on the results of the 3 subjective experiments for the groups divided by noise sensitivity (*L*_i,AFmax,_ (dB)).

Experiment	Noise Sensitivity	A	B	C	D	E	F
1st Low-BGN	High	26.6	34.2	41.7	49.2	56.8	64.3
	Low	26.7	34.9	43.1	51.2	59.4	67.6
2nd With BGN	High	28.1	36.0	44.0	52.0	60.0	68.0
	Low	28.0	36.1	44.3	52.4	60.5	68.7
3rd With BGN + Task	High	27.2	36.0	44.9	53.8	62.7	71.6
	Low	27.0	36.0	45.0	54.0	63.0	72.0

**Table 7:** Differences between the classification grade values for the sensitivity groups in the 3 experimental conditions.

Difference of Classification Grade Value between Two Sensitivity Groups (*L*_i,A,Fmax_ (dB))	A	B	C	D	E	F
1st Low BGN	0.1	0.7	1.4	2.0	2.6	3.3
2nd With BGN	−0.1	0.1	0.2	0.4	0.5	0.7
3rd With BGN + Task	−0.2	0.0	0.1	0.2	0.3	0.4

**Table 8:** Effects of background noise and additional tasks on the classification grade values for the sensitivity groups.

Difference of Classification Grade Value between Two Sensitivity Groups (*L*_i,A,Fmax_ (dB))		A	B	C	D	E	F
High sensitivity group	BGN effect	1.5	1.8	2.3	2.8	3.2	3.7
	Task effect	−0.9	0.0	0.9	1.8	2.7	3.6
Low sensitivity group	BGN effect	1.3	1.2	1.2	1.2	1.1	1.1
	Task effect	−0.1	0.1	0.2	0.4	0.5	0.7

**Table 9:** Results of the linear regression analysis for subjective responses in each of the 3 experimental conditions for groups divided by attitudes towards their neighbours.

Experiment	Attitude on Neighbours	Intercept	Slope	R-Square
1st Low-BGN	Not positive	−2.48525	0.13451	0.89086
	Positive	−2.32545	0.1153	0.8796
2nd With BGN	Not positive	−2.52999	0.12979	0.88484
	Positive	−2.29506	0.114	0.83974
3rd With BGN + Task	Not positive	−2.30621	0.11954	0.82902
	Positive	−1.72772	0.09953	0.8471

**Table 10:** Rubber ball impact sound classification grades calculated based on the results of the 3 subjective experiments for the groups divided by attitudes towards their neighbours (*L*_i,AFmax,_ (dB)).

Experiment	Attitude on Neighbours	A	B	C	D	E	F
1st Low-BGN	Not positive	25.9	33.3	40.8	48.2	55.6	63.1
	Positive	28.8	37.5	46.2	54.9	63.5	72.2
2nd With BGN	Not positive	27.2	34.9	42.6	50.3	58.0	65.7
	Positive	28.9	37.7	46.4	55.2	64.0	72.8
3rd With BGN + Task	Not positive	27.7	36.0	44.4	52.8	61.1	69.5
	Positive	27.4	37.5	47.5	57.5	67.6	77.6

**Table 11:** Differences between the classification grade values for the attitude groups in the 3 experimental conditions.

Difference of Classification Grade Value between Two Attitude Groups (*L*_i,A,Fmax_ (dB))	A	B	C	D	E	F
1st Low BGN	2.9	4.2	5.4	6.7	7.9	9.1
2nd With BGN	1.7	2.8	3.8	4.9	6.0	7.1
3rd With BGN + Task	−0.3	1.5	3.1	4.7	6.5	8.1

**Table 12:** Effects of background noise and additional tasks on the classification grade values for the attitude groups.

Difference of Classification Grade Value between Two Attitude Groups (*L*_i,A,Fmax_ (dB))		A	B	C	D	E	F
Not positive attitude group	BGN effect	1.3	1.6	1.8	2.1	2.4	2.6
	Task effect	0.5	1.1	1.8	2.5	3.1	3.8
Positive attitude group	BGN effect	0.1	0.2	0.2	0.3	0.5	0.6
	Task effect	−1.5	−0.2	1.1	2.3	3.6	4.8

**Table 13:** Results of the linear regression analysis for subjective responses in each of the 3 experimental conditions for the different structure types.

Experiment	Structure	Intercept	Slope	R-Square
1st Low-BGN	RC	−2.44235	0.13219	0.90275
	wooden	−2.25766	0.12827	0.94125
2nd With BGN	RC	−2.60029	0.12849	0.86908
	wooden	−2.55427	0.12086	0.92307
3rd With BGN + Task	RC	−2.29009	0.11897	0.85047
	wooden	−2.01516	0.1056	0.89072

**Table 14:** Rubber ball impact sound classification grades calculated based on the results of the 3 subjective experiments for the different structure types (*L*_i,AFmax,_ (dB)).

Experiment	Structure	A	B	C	D	E	F
1st Low-BGN	RC	26.0	33.6	41.2	48.7	56.3	63.9
	wooden	27.5	35.3	43.1	50.9	58.7	66.5
2nd With BGN	RC	28.0	35.8	43.6	51.4	59.2	66.9
	wooden	29.4	37.7	46.0	54.2	62.5	70.8
3rd With BGN + Task	RC	27.7	36.1	44.5	52.9	61.3	69.7
	wooden	28.6	38.0	47.5	57.0	66.4	75.9

**Table 15:** Differences between the classification grade values for different structure types in the 3 experimental conditions.

Difference of Classification Grade Value between Two Structures (*L*_i,A,Fmax_ (dB))	A	B	C	D	E	F
1st Low BGN	−1.5	−1.7	−1.9	−2.2	−2.4	−2.6
2nd With BGN	−1.4	−1.9	−2.4	−2.8	−3.3	−3.9
3rd With BGN + Task	−0.9	−1.9	−3.0	−4.1	−5.1	−6.2

**Table 16:** Effects of background noise and additional tasks on the classification grade values for the different structure types.

Difference of Classification Grade Value between Two Structures (*L*_i,A,Fmax_ (dB))		A	B	C	D	E	F
RC	BGN effect	2.0	2.2	2.4	2.7	2.9	3.0
	Task effect	−0.3	0.3	0.9	1.5	2.1	2.8
Wooden	BGN effect	1.9	2.4	2.9	3.3	3.8	4.3
	Task effect	−0.8	0.3	1.5	2.8	3.9	5.1

## Data Availability

Data available on request due to restrictions e.g., privacy.

## Appendix A

Questionnaire on noise sensitivity.

1. I wouldn’t mind living on a noisy street of the apartment I had was nice.
2. I am more aware of noise than I used to be.
3. No one should mind much if someone turns up his stereo full blast once in a while
4. At movies, whispering and crinkling candy wrappers disturb me.
5. I am easily awakened by noise.
6. If it’s noisy where I’m studying, I try to close the door or window or move someplace else.
7. I get annoyed when my neighbours are noisy.
8. I get used to most noises without much difficulty.
9. I don’t want to live across the fire station.
10. Sometimes noises get on my nerves and get me irritated.
11. Even music I normally like will bother me if I’m trying to concentrate.
12. It wouldn’t bother me to hear the sounds of everyday living from neighbours (footsteps, running water, etc.)
13. When I want to be alone, it disturbs me to hear outside noises.
14. I’m good at concentrating no matter what is going on around me.
15. In a library, I don’t mind if people carry on a conversation if they do it quietly.
16. There are often times when I want complete silence.
17. I think the sound from motorcycles is noisy.
18. I find it hard to relax in a place that’s noisy.
19. I get mad at people who make noise that keeps me from falling asleep or getting work done.
20. I wouldn’t mind living in an apartment with thin walls.
21. I am sensitive to noise.

Questionnaires on attitude toward neighbours

“How do you feel about your upstairs neighbours?”

1. They are good people.
2. I am happy to be their neighbour.
3. We understand each other in many things.
4. I know and understand their situation very well.
5. We greet each other with a friendly hello.
6. They try not to make as much noise as possible for us.

## Appendix B

Comparison of the regression method.

**Table A1:** Equations and R-square values of the regression methods.

Function	Equation	**R-Square**
Linear	y=0.1245x−2.54295	0.87815
Polynomial	$y=0.0016x^{2}-0.01627x-0.36436$	0.89731
Exponential	$y=1.93727exp\left(\frac{x}{43.6315}\right)-2.54655$	0.89528
Sigmoidal	$y=7.01647+\frac{\left(0.71291-7.01647\right)}{\left(1+exp\left(\left(x+51.45357\right)∕9.57445\right)\right)}$	0.90183
